# The role and potential mechanisms of LncRNA-TATDN1 on metastasis and invasion of non-small cell lung cancer

**DOI:** 10.18632/oncotarget.7788

**Published:** 2016-02-29

**Authors:** Niu Zequn, Zhang Xuemei, Li Wei, Ming Zongjuan, Zhong Yujie, Hou Yanli, Zhang Yuping, Meng Xia, Wang Wei, Deng Wenjing, Fan Na, Yang Shuanying

**Affiliations:** ^1^ Institute of Respiratory Medicine, The Second Affiliated Hospital, Xi'an Jiaotong University, Xi'an, China; ^2^ Institute of Pediatric Hematology and Oncology Medicine, Shanghai Oriental Hospital, Shanghai, China

**Keywords:** non-small cell lung cancer, LncRNA, TATDN1, invasion, metastasis

## Abstract

The invasion and metastasis of malignant tumor cells lead to normal tissue destruction and are major prognostic factors for many malignant cancers. Long non-coding RNA (LncRNA) is associated with occurrence, development and prognoses of non-small cell lung cancer (NSCLC), but its mechanisms of action involved in tumor invasion and metastasis are not clear. In this study, we screened and detected the expression of LncRNA in two NSCLC lines 95D and 95C by using high throughput LncRNA chip. We found that TATDN1 (Homo sapiens TatD DNase domain containing 1, TATDN1), one of LncRNAs, was highly expressed in 95D cells and NSCLC tumor tissues compared to 95C cells. Knockdown of TATDN1–1 by shRNA significantly inhibited cell proliferation, adhesion, migration and invasion in 95D cells. Further mechanism study showed that TATDN1 knockdown suppressed the expression of E-cadherin, HER2, β-catenin and Ezrin. Moreover, knockdown TATDN1 also inhibited tumor growth and metastasis in a 95D mouse model *in vivo* by inhibiting β-catenin and Ezrin. These data indicate that TATDN1 expression is associated with 95D cells' higher potential of invasion and metastasis, and suggest that TATDN1 may be a potential prognostic factor and therapeutic target for NSCLCs.

## INTRODUCTION

Lung cancer was the most commonly diagnosed cancer as well as the leading cause of cancer death, with 1.4 million deaths worldwide annually [1]. Almost 80% of lung cancers are non-small cell lung cancer (NSCLC) [2]. In males, the highest lung cancer incidence rates are in Eastern and Southern Europe, North America, Micronesia and Polynesia, and Eastern Asia [1]. Lung cancer surpassed breast cancer as the leading cause of cancer death in women and is expected to account for 26% of all female cancer deaths [3]. The prognosis for NSCLC is still dismal, and the overall 5-year survival is only 15% [4]. Treatment failure and death of the patients with NSCLC are correlated with high potential for invasion and metastasis. Therefore, identification of the predictive factors for invasion and metastasis of NSCLC is important to develop novel therapies for NSCLC.

Over 70% of the human genome is found actively transcribed, but only small proportion (1–2%) of the genome encodes proteins [5, 6]. LncRNAs are the main part of transcribed noncoding RNA [7]. Recent studies indicated that the type and amount of LncRNAs vary greatly among species, tissues and cells [8–11], and that they are biologically functional and associated with the development, progression and response to treatment of various diseases including cancer [12, 13]. LncRNAs have been found involve in gene expression regulation [11, 14], splicing [15], epigenetic control [16], chromatin structure [17, 18], and nuclear transport [11]. The following LncRNAs have been shown to be associated with the development and progression of lung cancer: HOTAIR, H19, ANRIL, MALAT1 (lung adenocarcinoma associated transcript 1) [19, 20], SCAL1 (smoke and cancer-related long-chain non-coding RNA1) [21], LncRNA AK126698 [22], and LncRNA GAS6-AS1 (GAS6 antisense RNA1) [23]. However, the role of LncRNA in NSCLC development, invasion and metastasis remains largely unknown.

In this study, we investigated the expression patterns of LncRNAs and mRNAs in 95D cell line, which is a human lung large cell lung cancer cell line with high metastatic potential, and in 95C cell line, which is a human lung large cell cancer cell line with low metastatic potential. We also investigated the effect of LncRNA-TATDN1 on tumor development, invasion and metastasis in high-expressed LncRNAs in 95D cells.

## RESULTS

### LncRNA TATDN1 was highly expressed in NSCLC 95D cells

**Figure 1:**
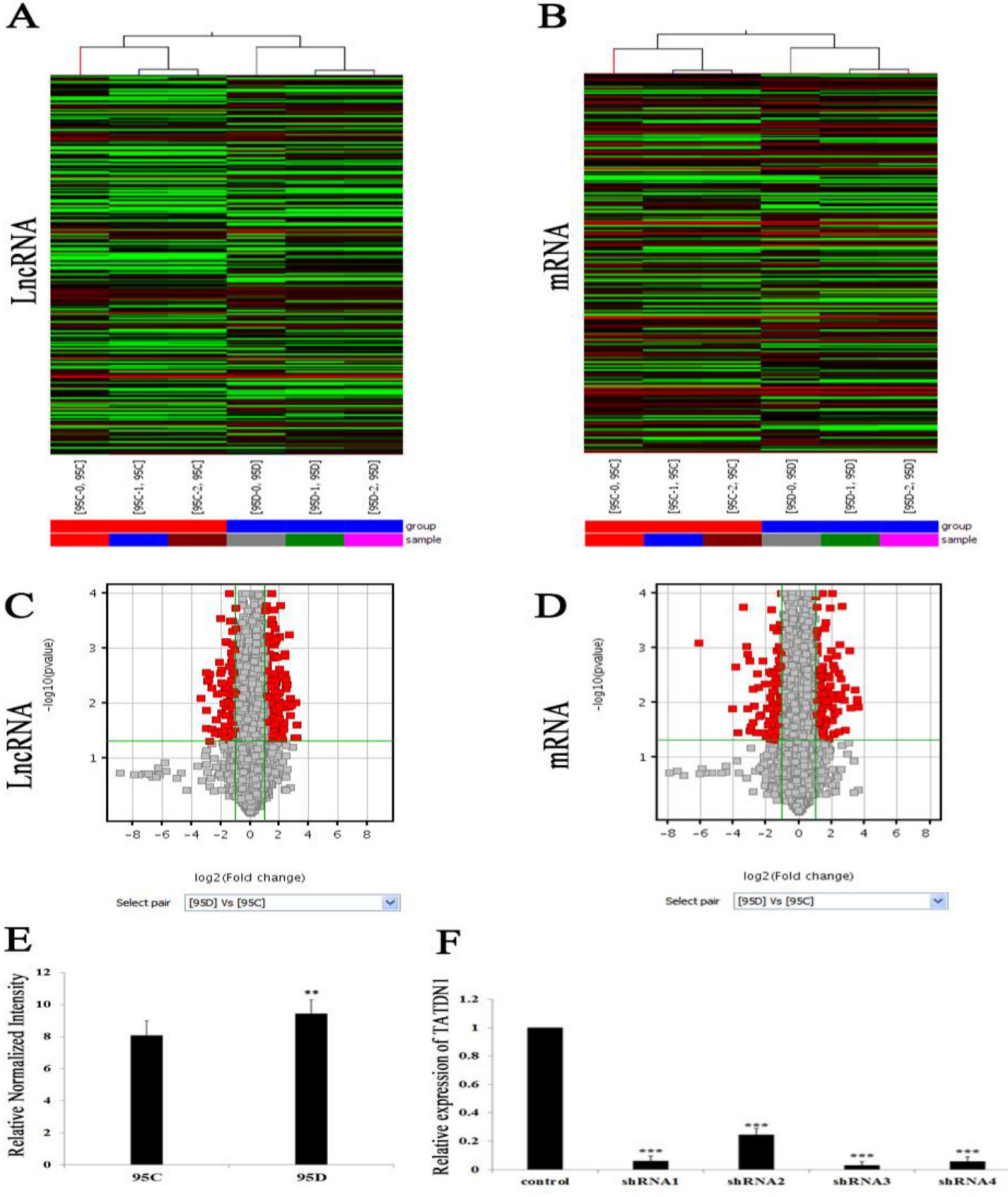
(**A–B**) Hierarchical clustering showed the expression differences of lncRNA and mRNA between 95D and 95C cells. (**C–D**) Volcano plot filtering showed the differences in LncRNA expression and mRNA between 95D and 95C cells. (**E**) TATDN1 was highly expressed in 95D cells than 95C cells. **p < 0.01. ShRNA3 was the strongest blocker (**F**), ***p < 0.001.

To investigate the potential biological functions of LncRNAs in NSCLC, we examined the lncRNA and mRNA expression profiles in human 95D and 95C cells through microarray analysis (Figure 1A–1D). The expression profiles of 313 LncRNAs and 252 mRNAs were found to be significantly different between the two cell lines. Among these, 198 LncRNAs were found to be upregulated more than two-fold while 115 LncRNAs were down-regulated more than two-fold in 95D cells than those in 95C cells. According to the expression difference in the level of LncRNA, the LncRNA TATDN1 was chose as an investigating target in this study. The fold change of LncRNA TATDN1 (FC = 2.5630, *p =* 0.0007) in 95D over 95C cell was found most significant (Figure 1E). When the 95D cell line was transfected with the lentivirus expressing TATDN1 siRNAs, the LncRNA TATDN1 was significantly blocked by the siRNAs and the silencing effect from the siRNA targeting at the site 3 is most significant (Figure 1F).

### TATDN1 knockdown suppressed cell proliferation, adhesion, invasion and migration in 95D cells

**Figure 2:**
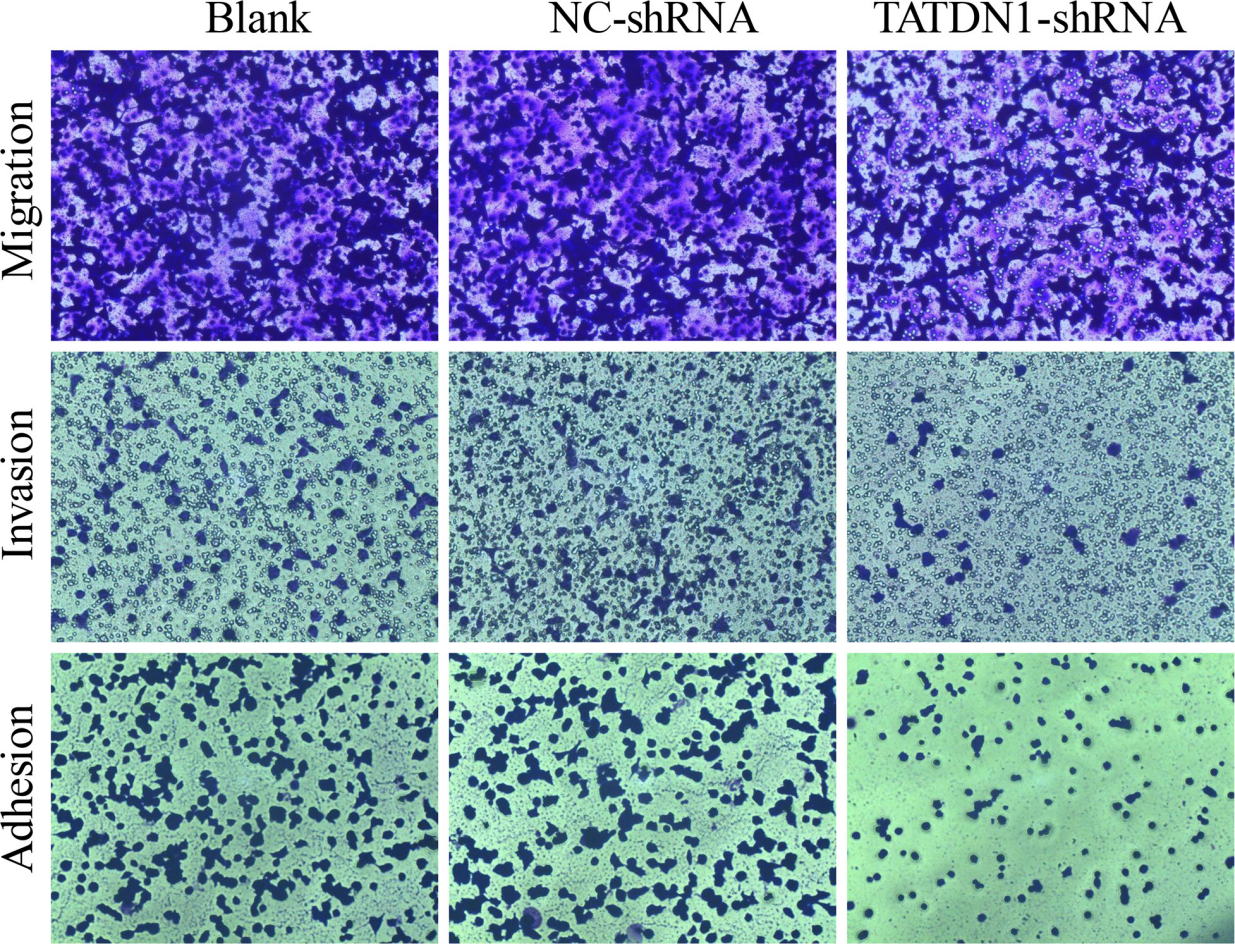
Effect of TATDN1 knockdown on cell proliferation, adhesion, invasion and migration The 95D cells were transfected with TATDN1 shRNA or NC-shRNA, and cell proliferation, adhesion, invasion and migration were detected.

To assess the biological role of TATDN1 in 95D cells, we blocked the expression of TATDN1 in 95D cell and determined the effect of TATDN1 on cell proliferation by MTT. The results showed that knockdown of TATDN1 significantly inhibited the proliferation of 95D cells transfected with pGMLV-SC5 compared to the negative controls (Figure 2).

Cell invasion is a significant aspect of cancer progression and involved in the migration of tumor cells into contiguous tissues and the dissolution of extracellular matrix proteins. To examine whether TATDN1 has a direct role in facilitating 95D cells adhesion, migration and invasion, we evaluated the effect of TATDA1 inhibition on cell adhesion and invasion by Matrigel and on migration by transwell. As shown in Figure 2, inhibition of TATDN1 impeded the adhesion, invasion and migration of 95D cells compared to the control group. These data indicate that TATDN1 could promote the migratory and invasive phenotype of 95D cells.

### TATDN1 knockdown inhibited cell motion capability in 95D cells

**Figure 3:**
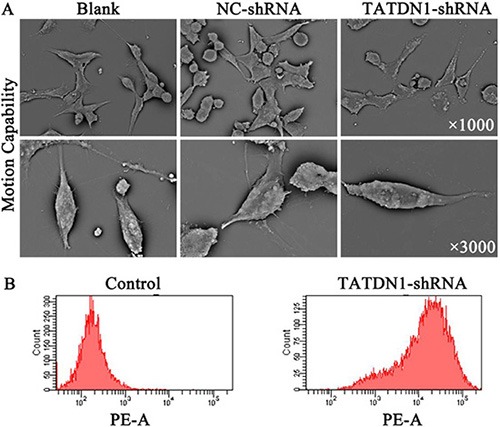
Effect of TATDN1 knockdown on the expression of metastasis-related factors and E-cadherin Pseudopodium, filamentous cilia (1000 ×) and microvillus (3000 ×) were detected by SEM in 95D cells with Lnc-TATDN1 knocked down (**A**). The expression of E-cadherin in TATDN1 shRNA 95D cells was detected by Flow cytometry (**B**).

We next studied the effect of TATDN1 inhibition on motion capability change of 95D cells by a scanning electron microscope (SEM). At a magnification of 1.0 K ×, we detected shrinking cell morphology, shorter and thinner filopodia, and the reduced cell number in the TATDN1-shRNA transfected-95D cells. Moreover, at a more detailed micrograph at 3.0 K ×, we observed the visible cell surface, the smooth, projections and the decreased microvillius in the TATDN1-shRNA transfected-95D cells (Figure 3A).

### TATDN1 knockdown reduced E-cadherin expression in 95D cells

E-cadherin has been shown to participate in the development and architectural maintenance of epithelial tissues and has signaling capabilities [24], which is dysregulated and down-regulated in lung cancer [25]. We next detected the effect of TATDN1 on E-cadherin expression in 95D cells by flow cytometry analysis. The result showed that knockdown of TATDN1 increased the expression level of E-cadherin on 95D cell membrane (Figure 3B).

### TATDN1 knockdown upregulated Nm23-H1 and inhibited HER2 mRNA expression in 95 D cells

**Figure 4:**
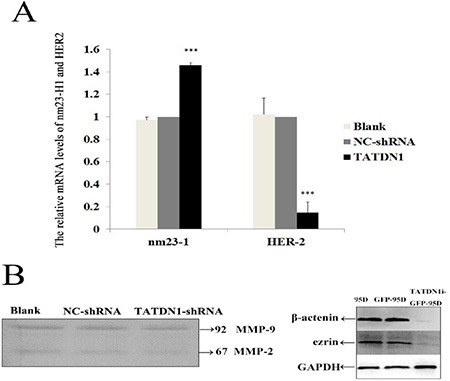
Effect of TATDN1 knockdown on the expression HER2, MMP2/9, β-catenin and ezrin in 95D cells (**A**) HER2 mRNA level was detected by RT-PCR analysis ***p < 0.001. (**B**). The active MMP-2 and MMP-9. (B) β-catenin and ezrin proteins were detected by Western bolt analysis.

Human epidermal growth factor receptor 2 (HER2) dimerization initiates a variety of signaling pathways leading to cell proliferation and tumorigenesis. The metastatic suppressor nm23 gene family is highly conserved among a wide variety of eukaryotic species [26]. To further explore the underlying mechanism of TATDN1 in the migration process in 95D cells, we investigated the effect of TATDN1 on HER-2 and nm23-H1 expression by RT-PCR. The results showed that knockdown of TATDN1 increased the mRNA level of nm23-H1 but decreased HER2 levels in 95D cell (Figure 4A).

### TATDN1 knockdown suppressed β-catenin and Ezrin expression in 95D cells

MMP2/9, β-catenin and ezrin drives tumor progression and metastasis of cancer [27–29]. We also examined the effect of TATDN1 on MMP2, MMP-9 activity and the expression of β-catenin and ezrin proteins in 95D cells. As shown in Figure 4B, the activity of MMP2, MMP-9 did not change significantly. As expected, down-regulation of TATDN1 in 95D cells resulted in the decrease of β-catenin and Ezrin protein expression (Figure 4B). These data indicate that TATDN1 may influence the invasive and metastatic potential of NSCLC cells by altering β-catenin and Ezrin protein expression.

### TATDN1 knockdown inhibited tumor growth and metastasis by regulating β-catenin and Ezrin in a 95D mouse model

**Figure 5:**
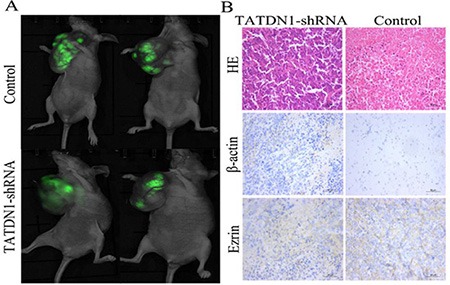
Effect of TATDN1 knockdown on tumor growth and metastasis in a 95D mouse model The mice were injected with the 95D cells transfected with shRNA against TATDN1. The tumor growth were measured by MicroSPECT analysis (**A**). The expression levels of β-catenin and ezrin protein were analyzed by IHC (**B**).

To validate the effect of TATDN1 on the growth of metastasis of 95D cells *in vivo*, 12 male Balb/c–nude mice (4–5 weeks–old) were subcutaneously injected with TATDN1-shRNA or NC-shRNA cells in 150 μl of PBS in each right armpit. The mice were euthanized by cervical vertebra dislocation at the end of the experiments. The animal studies were approved by the local Ethics Committee (Xi'an Jiao Tong University, School of Medicine) and performed according to ethical principles of animal experimentation. the control NC-shRNA cells. After 2 months, the animals were sacrificed for determination of tumor dimension, and the tumors were subjected to immunohistochemical analysis. As showing in Figure 5A, TATDN1 knockdown effectively inhibited tumor growth and metastasis. Immunohistochemical analysis revealed lower expression of β-catenin and Ezrin proteins in the cells transfected with TATDN1-shRNA compared to the control groups (Figure 5B). These *in vivo* data further demonstrated that TATDN1 was capable of regulating 95D cell growth and metastasis *in vivo*.

## DISCUSSION

The prognosis for NSCLC is still dismal, and the overall 5-year survival of only 15% [4]. The most important reason for treatment failure and death in patients with lung cancer is lung cancer invasion and metastasis. Metastasis is the end product of an evolutionary process in which diverse interactions between cancer cells and their microenvironment yield alterations that allow these cells to transcend their programmed behavior. Tumor cells thus populate and flourish in new tissue habitats and, ultimately, cause organ dysfunction and death [30]. A deeper understanding of the molecular and genetic concepts and processes involved in lung cancer metastasis may pave the way toward new prognostic models and ways of planning treatment.

With a rapid development of molecular biology, LncRNA has caused a great concern and becomes the hot issue and frontier because of the specialty of its function. LncRNAs are crucial for maintaining cellular physiology, and disequilibrium in the balance of lncRNAs could significantly contribute to the onset and development of several pathologies. For example, *MALAT-1* has been primarily studied for its role in cancer cells migration, invasion and metastasis [20, 31]. In addition, it plays an important role in cell cycle progression. However, there are few reports on the role which LncRNA plays in tumorigenesis mechanism. In our study, We detected differentially expressed LncRNA between 95D and 95C cells and found high levels of LncRNA TATDN1 in 95D cells. The full name of TATDN1 is Homo sapiens TatD DNase domain containing 1. It is locus NR_027427, GI:225903440, FC was 2.56 times, *P* < 0.001, length of 1158bp, located at chromosome 8. It is located near the ring finger protein related gene 139 (Ring finger protein139, RNF139). We next srudied the detailed role of TATDN1 in the regulation of metastasis in NSCLC cells.

Metastasis, the spread of tumour cells from their primary sites to secondary sites within the body, is a multiple-step process that requires the accumulation of altered expression of many different genes. This complex process involves cell adhesion, degradation of the surrounding extracellular matrix, migration, proliferation at a secondary site, and stimulation of angiogenesis [32, 33]. We found knockdown of TATDN1 by shRNA significantly suppressed cell proliferation in 95D cells *in vitro*. In addition, we also confirmed the effect of TATDN1 on cell growth in a mouse model *in vivo*.

In many primary tumors with invasive properties, intercellular adhesion is reduced because of a loss of E-cadherin, a direct mediator of cell–cell adhesive interactions. The cytoplasmic tail of E-cadherin is tethered, via α-catenin and β-catenin, to the actin cytoskeleton; one of actin's properties is to maintain cell junctions. The importance of maintaining intercellular adhesion was shown in a mouse model of pancreatic cancer in which disruption of the expression of E-cadherin led to early invasion and metastasis [34]. β-Catenin was expressed in 94% of resected squamous cell lung cancer samples and in 51% of adenocarcinomas, and β-catenin overexpression was associated with improved prognosis [28]. Knockdown of ezrin by using small interfering RNA (siRNA) resulted in significant reduction in proliferation, migration and invasion of these cells *in vitro*. In this study, we confirmed that knockdown of TATDN1 in 95D cells might increase significantly the expression level E-cadherin, while β-catenin and ezrin protein was decreased. Epithelial–mesenchymal transition (EMT) is a critical role of tumor invasion and metastasis, and E-cadherin is an important symbol to reduce the occurrence of EMT. ECM has been found that almost all of the ingredients can be degraded by MMPs, so that the epithelial cells - and cells - connection between matrix is destroyed, decreased adhesion of epithelial cells, more off and leave the site where the occurrence invasion and metastasis. The results showed that the activity of MMP-2 and MMP-9 had no significant changes between the experimental group and the control group.

In murine models, activation of Wnt signaling is associated with increasing tumor initiation potential [35], which plays an important role in the development of NSCLC. β-catenin is the hub of molecular wnt signaling pathway, mediated wnt signals from the cell membrane to the cytoplasm into the nucleus transfer. Increasing extracellular matrix metalloproteinase inducer (emmPrIN) levels in lung cancer cells upregulated the β-catenin signaling pathway, and silencing emmPrIN inhibited β-catenin signaling, cell migration, proliferation, anchorage independent growth, and xenograft growth [36]. Ezrin primarily acts as a linker between the plasma membrane and the cytoskeleton and is a key component in tumor metastasis. It has been reported that silencing of ezrin induced an increased E-cadherin expression and a decreased phosphorylation of beta-catenin by inhibiting phosphorylation levels of c-src [37]. The PI3K/AKT/MTOR pathway is an important signaling cascade in many different types of human cancer. This pathway has been linked to cell survival, differentiation, proliferation, growth, metabolism, migration, and angiogenesis. Under normal physiological conditions, the phosphorylation of ezrin Y353 is important for survival of epithelial cells through activation of the PI3K/Akt pathway, and 3D cell cultures of the epithelial cell line LLC-PK1 showed that ezrin binds to p85, the regulatory subunit of PI3K, in order to mediate the PI3K/Akt pathway [38]. Therefore, overexpression of ezrin as a result of Myc over expression could, in turn, maintain the survival of cancer cells through activation of the PI3K/Akt pathway. In our *vitro* and *vivo* study, we found that silencing of TATDN1 by using shRNA resulted in significant reduction in β-catenin and Ezrin protein. According to our findings, it speculates that TATDN1 may mediate 95D cells by Wnt/β-catenin signal pathways and PI3K/AKT/MTOR pathway, thus β-catenin and Ezrin is respectively effect target of the LncRNA.

In summary, our results demonstrate that TATDN1 is an important LncRNA in NSCLC and it overexpresses in 95D cells. Inhibition of TATDN1 suppressed cell invasion and migration ability through the inhibition of β-catenin, Ezrin and HER2 expression. Our findings have shown the potential of TATDN1 in regulating lung cancer metastasis, and that the Wnt/β-catenin signal pathways and PI3K/AKT/MTOR pathway may be involved in the key mechanism of its anti-metastatic effect. LncRNA TATDN1 may provide new molecular biomarkers for the diagnosis of NSCLC.

## MATERIALS AND METHODS

### Cell culture

All the cells were cultured in the RPMI1640 medium (from Roswell Park Memorial Institute, Hyclone, USA) with 10% fetal calf serum, 100 U/mL penicillin, and 100 μg/mL streptomycin, at 37°C, high humidity, and 5% CO2. (The cell lines was obtained from Chinese Academy of Science).

### RNA extraction and LncRNA microarray

Total RNA was extracted from cells using TRIzol reagent (Invitrogen, USA) following the manufacturer's instruction, and purified by RNeasy Mini Kit (from Qiagen). Total RNA from each sample was quantified. RNA integrity was assessed using standard denaturing agarose gel electrophoresis, and the purity was judged by the ratio of absorbance at 260 nm to 280 nm (A260/A280).

The sample preparation for microarray hybridization followed performed based on the manufacturer's standard protocols with minor modifications. Briefly, mRNA was purified using mRNA-only Eukaryotic mRNA Isolation Kit (from Epicentre). Then, each sample was amplified and transcribed into fluorescent cRNA along the entire length of the transcripts without the 3′ bias, utilizing a random priming method. The labeled cRNAs were hybridized onto the Human LncRNA Array v2.0 (8 × 60 K, Arraystar). After washing the slides, the arrays were scanned by the Agilent Scanner G2505B. According to the position of gene, gene chip fluorescence intensity, *P* values and fold change (FC) values, purposive lncRNA was selected for further study.

### Lentivirus construction and target screening for RNAi

Four sequences (sites 1, 2, 3, and 4) of the TATDN1 gene were selected as the target for RNA interference (RNAi) (Jiman, Shanghai, China). The sequence of site 1 was 5′-GCACTGCATTTGGCACAAACA-3′, the sequence of site 2 was 5′-GGATGTCATCCTACAAG.

ATGT-3′, the sequence of site 3 was 5′-GGGAGT GGTGCATTCATTTGA-3′, the sequence of site 4 was 5′-GGGAAAGTTGTGGCAATAGGA-3′. The control non-coding (NC) sequence is 5′-TTCTCCGAACGTGT CACGT-3′. Then the pGMLV-SC5RNAi vectors containing the TATDN1 RNAi sequences were constructed. A lentivirus was produced by co-transduction of the siRNA expression pGMLV-SC5RNAi vectors into the 293T cells. Then selected lentiviruses containing integrated TATDN1 RNAi sequences were used to transfect 95D cells. Use PCR method tofind most effective gene interference in 95D cells with non-lentivirus and lentivirus containing NC-shRNA transduction as controls.

### Cell proliferation assay

The TATDN1-shRNA-95D cells, NC-shRNA-95D cells and 95D-cells were grown to a density of 8 × 10^3^ cells per well in a flat bottomed 96-well plate. All cells were cultured for 24, 48, and 72 hours respectively, followed byincubating with 20 μL MTT solution (from Sigma, USA) for 4 hours. Then, the cells were exposed to DMSO (from Gibco, USA). As a blank control, 200 μL 10% FBS was used. The optical density (OD) was measured using an absorbance microplate reader (from Bio-Tek, USA) at a wavelength of 490 nm. The cell proliferation was expressed as a percentage of the OD value of the controls.

### Cell adhesion assay

We coated 96-well plates with Matrigel (from BD, USA) for 1 hour. The TATDN1-shRNA-95D cells, NC-shRNA-95D cells and 95D cells (8 × 10^4^) were dispensed into the 96-well plates and then were incubated for 2 h. The well were washed 3 times with PBS and viable cells were determined.

### Transwell assay

The invasive activity of TATDN1-shRNA-95D, NC-shRNA-95D and 95Dcells was assessed using 24-well transwell insert coated with Matrigel. The cells (2 × 10^5^) in serum-free medium were seeded into the upper chamber. Medium supplemented with 10% fetal bovine serum was placed in the lower chamber as chemo-attractants. After 48 hours of incubation, the cells on the upper surface of the filter were removed with a cotton swab, and cells, which invaded through the Matrigel and were adherent to the lower surface of the filter, were fixed and stained with 0.5% crystal violet and counted under a light microscope.

### Electron microscopy assay

The climbing parts of above cultured TATDN1-shRNA-95D, NC-shRNA-95D and 95D cells were fixed with a solution 2.5% glutaraldehyd and then used 1% Osmic acid fixation, After dehydration, thin sections were stained with uranyl acetate and lead citrate for observation under an electron microscope (from Hitachi, Japan). Digital images were obtained using an Advanced Microscopy Techniques imaging system.

### Flow cytometry assay

The TATDN1-shRNA-95D and 95D-cells were detached using trypsin-EDTA (from Hyclone, USA), followed by inactivating the enzyme by adding 10% FBS RPMI-1640. After washed triple using PBS, the cell density was adjusted to 1 × 10^6^ /ml in100 μl of absorbent with 5 μl of E-cadherin antibody under dark condition (to avoid light exposure). The cell suspension was incubated at 4°C for 30 min, washed 3 times, and adjusted to 400 μl. Then the cell suspension was examined by flow cytometry (FACSCanto II, from BD, USA).

### Quantitative PCR assay

Total RNA was extracted from cells using RA2 reagent (from Kangwei, China) following the manufacturer's instruction, and purified by RNeasy Mini Kit (from Qiagen). Total RNA from each sample was quantified and quality-assured by NanoDrop ND-1000. To analyze the of nm23-H1 and c-erbB-2 inTATDN1-shRNA-95D, NC-shRNA-95D, and 95D cells, nm23-H1 and c-erbB-2 primers were designed as nm23-H1 sense is5′-TTAATCAGATGGTCGGGGAT-3′; the nm23-H1 antisense was 5′-GATCTATGAATGACAGGAGG-3′; the c-erbB-2 sense was 5′-AGGGCAGTTACCAGTGCCAAT ATC-3′, the c-erbB-2 antisense was 5′-TCCAGAGTCTC AAACACTTGGAGC-3′. The reaction was carried out in a final volume of 25 μl containing 5 μl of RT reaction. Negative control reactions were performed to rule out any contamination. The thermal cycling profile was the same for each primer set and consisted of an initial denaturation at 95°C for 10 min, followed by 40 amplification cycles of 20 seconds at 55°C, and 15 seconds at 72°C. Melting curve analysis was used to confirm the specificity of the amplification products. GAPDH was the housekeeping gene used as an internal primer control. A positive cDNA control was also used as reference (the isoform A, the most abundantly expressed).

### Zymography assay

Gelatin zymography was performed to determine the activity of MMP-2 and MMP-9. Protein quantitative by NanoDrop ND-1000 assay. Briefly, the protein in medium was separated in 10% SDS-PAGE gel containing 1 mg/ml gelatin at 4°C. The protein levels of MMP-2 and MMP-9 were evaluated by zymography assay.

### Western-blot assay

Whole cell lysates from the cultured cells were harvested with cell lysis buffer. Equal amounts of the protein (20 μg) were separated by SDS-PAGE, and transferred to a polyvinylidene difluoride membrane (Millipore, Bedford, MA, USA). The membrane was blocked with 5% skimmed milk in Tris-buffered saline and then incubated with the primary antibody for 1 hour at room temperature, and after washing incubated with second antibody for 1 hour. After washing 3 times the membrane was incubated with ECL mixture and exposed in a dark room.

### Tumor xenograft

Mice were housed and maintained in laminar airflow cabinets under specific pathogen-free conditions. TATDN1-shRNA-95D and NC-shRNA-95D cells were harvested from culture flasks and transferred to serum-free PBS. Single-cell suspensions (1e10^7^ in 200 μL) were injected subcutaneously into the right armpit of female Balbc nude mice (4–5 weeks old) from the animal laboratory of Xi'an Jiao Tong University (Xi'an, China). Tumor size was evaluated using a standard caliper by a person who was blind of the experimental group. After 2 months of xenografting, the tumor was removed. For histological analyses, hematoxylin and eosin staining was performed. Immunohistochemical staining was performed using an antibody cytokeratin (βsing an antibodyEzrin CST 1:100). All experimental protocols were reviewed and approved by the Ethic Committee on Animal Experimentation in Xi'an Jiao Tong University.

### Statistical analyses

Data are expressed as the mean values ± standard deviation from at least three experiments. Statistical comparisons were based on Student's *t*-test or analysis of variance. *P* < 0.05 was considered to indicate a statistically significant difference.
